# Ischemia as a possible effect of increased intra-abdominal pressure on central nervous system cytokines, lactate and perfusion pressures

**DOI:** 10.1186/cc8908

**Published:** 2010-03-15

**Authors:** Athanasios Marinis, Eriphili Argyra, Pavlos Lykoudis, Paraskevas Brestas, Kassiani Theodoraki, Georgios Polymeneas, Efstathios Boviatsis, Dionysios Voros

**Affiliations:** 1 Second Department of Surgery, Aretaieion University Hospital, 76 Vassilisis Sofia's Av, GR-11528, Athens, Greece; 2 First Department of Anesthesiology, Aretaieion University Hospital, 76 Vassilisis Sofia's Av., GR-11528, Athens, Greece; 3 Department of Neurosurgery, "Evangelismos" Athens General Hospital, 45-47 Ipsilantou STR, GR-10676, Athens, Greece

## Abstract

**Introduction:**

The aims of our study were to evaluate the impact of increased intra-abdominal pressure (IAP) on central nervous system (CNS) cytokines (Interleukin 6 and tumor necrosis factor), lactate and perfusion pressures, testing the hypothesis that intra-abdominal hypertension (IAH) may possibly lead to CNS ischemia.

**Methods:**

Fifteen pigs were studied. Helium pneumoperitoneum was established and IAP was increased initially at 20 mmHg and subsequently at 45 mmHg, which was finally followed by abdominal desufflation. Interleukin 6 (IL-6), tumor necrosis factor alpha (TNFa) and lactate were measured in the cerebrospinal fluid (CSF) and intracranial (ICP), intraspinal (ISP), cerebral perfusion (CPP) and spinal perfusion (SPP) pressures recorded.

**Results:**

Increased IAP (20 mmHg) was followed by a statistically significant increase in IL-6 (*p *= 0.028), lactate (*p *= 0.017), ICP (*p < 0.001*) and ISP (*p *= 0.001) and a significant decrease in CPP (*p *= 0.013) and SPP (*p *= 0.002). However, further increase of IAP (45 mmHg) was accompanied by an increase in mean arterial pressure due to compensatory tachycardia, followed by an increase in CPP and SPP and a decrease of cytokines and lactate.

**Conclusions:**

IAH resulted in a decrease of CPP and SPP lower than 60 mmHg and an increase of all ischemic mediators, indicating CNS ischemia; on the other hand, restoration of perfusion pressures above this threshold decreased all ischemic indicators, irrespective of the level of IAH.

## Introduction

Intra-abdominal hypertension (IAH) and abdominal compartment syndrome (ACS) are two clinical entities constituting a continuum of pathophysiologic sequelae ranging from mild elevations of intra-abdominal pressure (IAP) to the devastating effects of organ hypoperfusion and, uneventfully, to death. Although effects of increased intra-abdominal pressure (IAP) on various organs and systems have been reported over 150 years ago, pathophysiologic implications have been rediscovered and definitions and recommendations developed the last few years [1-7].

Currently, the pathophysiological interaction of the abdominal compartment with other compartments (thoracic, cranial and extremities) has been extensively studied, comprising the polycompartment syndrome, a term coined by Malbrain M et al [5,8]. This holistic view emphasizes the relationships developing directly and indirectly between these body compartments, with potential therapeutic implications in everyday practice.

An interesting part of this concept is the relationship of IAH and the central nervous system (CNS) [9]. Several experimental and clinical studies have described the development of intracranial hypertension (ICP) and the decrease in cerebral perfusion pressure (CPP) during IAH [10-21]. These findings are based upon the modified Monroe-Kellie doctrine which recognizes four main contents in the cranial space (osseous, vascular, cerebrospinal fluid and parenchyma) the volume of each reciprocally affecting each other. Moreover, Bloomfield et al [13-15] suggested a mechanical effect of elevated IAP on CNS; IAH raises intrathoracic pressure (ITP) and jugular venous pressure, impeding cerebral venous outflow. This results in an increase of the vascular component and leads to increased ICP. Another mechanism was proposed by Halverson et al [16], who demonstrated experimentally that increased ICP during pneumoperitoneum was caused partially by decreased absorption of the cerebrospinal fluid (CSF) in the region of the lumbar cistern and the dural sleeves of the spinal nerve roots. He suggested that this finding correlates with the effect of increased inferior vena cava pressure on the lumbar venous plexus, the outflow of which is restricted, further impeding CSF absorption from the arachnoid villi.

Taking into consideration the current knowledge concerning the impact of IAH on the CNS we conducted an experimental study in animals in order to investigate whether increased ICP and intraspinal pressure (ISP), as well as decreased CPP and spinal perfusion pressure (SPP), might possibly lead further to CNS ischemia. Development of ischemia was demonstrated by changes in the CSF concentration of interleukin 6 (IL-6), tumor necrosis factor alpha (TNFa) and lactate, which are considered to increase when CNS ischemia ensues [22-35].

## Materials and methods

The study was performed in the experimental laboratory 'Kostas Tountas' of the Second Department of Surgery at the Aretaieion University Hospital (Athens School of Medicine, National and Kapodistrian University of Athens), conforms to our institutional standards and is under the appropriate license of the veterinary authorities and in adherence to National and European regulations for animal studies.

The protocol of our experimental study enrolled 15 female pigs (*Sus scrofa domesticus*) with a mean weight of 30 kg (range, 25 to 35 kg). The first three animals were a priori decided to be sacrificed in order to develop and standardize our protocol. The corresponding data were not complete so the animals were excluded from our data. All animals were fasted for 12 hours before the experiment, with free access to water.

### Anaesthesia

Sedation was achieved by intramuscular injection of ketamine (4 to 6 mg/kg), atropine (0.5 mg/kg), and midazolam (0.75 mg/kg). Then an intravenous line was placed in the greater auricular vein and general anaesthesia was induced by thiopental 5 mg/kg, and fentanyl 2 μg/kg, and the animal was intubated. Basic monitoring (electrocardiogram, oxygen saturation, non-invasive pulse and arterial pressure monitoring) was applied. Anaesthesia was maintained by isoflurane 0.5 to 1.5%, vecuronium 0.1 mg/kg/h, fentanyl 2 μg/kg and midazolam 5 mg/h. The animals were ventilated mechanically (Drager Sulla 808V, type Ventilog-2, Drager, Berlin, Germany) in a mixture of N_2_O/O_2 _at a FiO_2 _0.4 to 0.6, respiratory rate varying from 16 to 30 breaths per minute and tidal volumes ranging between 450 to 600 ml, aiming at an end-tidal CO_2 _= 35 to 45 mmHg. End-tidal concentration of N_2_O and isoflurane was monitored continuously throughout the study in order to ensure that depth of anaesthesia was maintained and boluses of 25 to 50 μg fentanyl and 5 mg midazolam were administered according to needs.

Fluid infusion rate was standardized at 5 ml/kg/h during pneumoperitoneum and was modified to 10 ml/kg/h after abdominal desufflation.

### Instrumentation

An 18 G catheter (Portex^® ^minipack system, Smiths Medical, Dublin, OH, USA) was placed after lumbar puncture in the subarachnoid space and the correct placement was confirmed by the aspiration of CSF. The catheter was connected through a non-compressible tubing system to a standard transducer. Calibration was performed using the right atrium as a zero point ensuring that the operative table was in a neutral position. ISP pressures were measured and CSF samples (0.5 to 1 ml each) were collected through this catheter.

A burr hole (2.7 mm) at a point 2 cm above the animal's eyebrow served as a pathway for introducing intracerebrally the Codman ICP monitoring system^® ^(Johnson & Johnson, Raynham, MA, USA), which includes a transducer (Codman Microsensor Transducer) that connects to the pressure monitoring system (Codman ICP express). Calibration was performed with the animal in a neutral position, according to manufacturer's instructions. ICP pressures were measured through this system.

After surgical right neck dissection, the neurovascular bundle was exposed and a 20 G catheter (Arterial Leader-Cath 115.090, Vygon Corporation, Montgomeryville, PA, USA) was introduced in the carotid artery for invasive blood pressure monitoring and blood collection. Then an introducer sheath 6 to 6.5 French was placed in the ipsilateral internal jugular vein in order to pass through it a Swan-Ganz catheter 5.5 Fr (Pediatric Oximetry Thermodilution Catheter, model 631HF55, Edwards Lifesciences, Irvine, CA, USA). The placement of the catheter in the pulmonary artery was conformed and calibrated.

A single lumen venous catheter (Leader-Cath 15 cm 119, Vygon Corporation, Montgomeryville, PA, USA) was introduced into the inferior vena cava via the femoral vein for collecting blood for cytokine measurement and recording inferior vena cava pressure (IVCP).

### Experimental phases

After instrumentation, animals were stabilized for 45 to 60 minutes (baseline phase T1). Then hemodynamic parameters and CNS pressures were recorded and CSF and blood samples collected. The next phase (T2) started with the introduction of a Veress needle through a small horizontal infra-umbilical incision into the peritoneal cavity. After connecting the Veress needle to the laparoscopic insufflator, a preset IAP of 20 mmHg was established mimicking intra-abdominal hypertension grade II. Helium was used for insufflation instead of CO_2 _in order to eliminate effects on blood gases [36]. IAP of 20 mmHg was maintained for 45 to 60 minutes and then pressures were recorded and samples collected as in phase T1. Phase T3 included a further rise of IAP by establishing a pneumoperitoneum of 45 mmHg for another 45 to 60 minutes, mimicking ACS, after which pressures were recorded and samples collected. Finally, the abdomen was desufflated by opening the Veress needle to the air (phase T4). After 45 to 60 minutes of animal stabilization, pressures were recorded and samples collected.

Induction of IAP in this animal study was clearly mechanical, without developing conditions either of capillary leakage which could interfere in interpretation of cytokines and lactate measurements or intravascular depletion (e.g. hemorrhage) interfering with hemodynamic measurements. A net effect of mechanically increased IAP on CNS pressures, cytokines and lactate was attempted. We didn't use a gradual increase of IAP, but rather a first level of 20 mmHg, commonly seen in clinical settings, and then an abrupt increase to 45 mmHg, in order to augment the impact of IAH on CNS and draw safer conclusions for this relationship. Finally, the increase of IAP in phase T3 is considered as ACS, according to definitions of the World Society of the Abdominal Compartment Syndrome (WSACS)[1].

### Calculation of preload assessment parameters

It is well established that increased IAP increases ITP mechanically by the cephalad elevation of the diaphragm, simultaneously affecting preload intracardiac filling pressures used traditionally, such as central venous pressure (CVP), pulmonary arterial occlusion pressure (PAOP), left atrial pressure and left ventricular end-diastolic pressure. This phenomenon, called abdomino-thoracic transmission, has been well studied in several reports and resumed in an excellent editorial by Malbrain et al [8] and is considered to be 50%. Currently, preload assessment during IAH and ACS is accomplished by using volumetric indices, such as right ventricular end-diastolic volume (RVEDV), global end-diastolic volume (GEDV) and stroke volume variation (SVV). However, when these cannot be used for practical reasons the calculation of transmural pressures can be used instead:

Transmural PAOP = PAOP - IAP/2 and Transmural CVP = CVP - IAP/2 [37].

### Calculation of abdominal and CNS perfusion pressures

Abdominal (APP), cerebral (CPP) and spinal (SPP) perfusion pressures were calculated by subtracting IAP, ICP and ISP from mean arterial pressure (MAP) respectively:

APP = MAP - IAP

CPP = MAP - ICP

SPP = MAP - ISP

### Measurement of cytokines and lactate

Cytokines were measured by the ELISA technique, using: a) the porcine ELISA test kits for IL-6 and TNFa (Assay-design, Ann Arbor, MI, USA) for measurements in CSF samples and b) the porcine ELISA test kits for IL-6 and TNFa (Hyucult Biotech, Uden, Netherlands) for the same purpose in blood samples. Lactate was measured with a portable analyzer (Lactate Scout analyzer, Sports Resource Group, Inc., USA).

### Study end-points

The main aim of this study was to demonstrate the development of CNS ischemia under conditions of high IAP. Two key elements were investigated as ischemic predictors: decrease in CNS perfusion pressures and increase of ischemia mediators (IL-6, TNFa and lactate).

Secondary end-points were the evaluation of the impact of IAH on ICP and ISP, cardiovascular, respiratory and acid-base homeostasis.

### Statistical analysis

Parametric and non-parametric tests were used according to the distribution of measurements (tested by the Anderson-Darling normality test). Thus, measurements of all indicators in the blood and CSF are displayed as median ± interquartile range (IQR), while measurements of pressures are displayed as mean ± standard deviation, respectively. Analysis of variance was applied using the Friedman test. Wilcoxon paired-ranks test was used for comparison of TNFa, IL-6 and lactate, while the paired t-test was used for comparison of CNS pressures. Relationships and covariance between variables were investigated by Spearman correlation coefficient analysis. The statistical software SPSS 11.0 (SPPS Inc., Chicago, IL, USA) was used.

## Results

With the exception of one animal (pig #5 died after pneumoperitoneum was induced due to a massive pulmonary embolism and was excluded from the study), 11 animals were included for the analysis of experimental data, which are as follows.

### Cytokines (Table 1)

**Table 1:** Changes of concentrations ofCNS ischemia indicators during thefour experimental phases (T1 to T4).

	T1 (baseline)		T2 (IAP = 20 mmHg)		T3 (IAP = 45 mmHg)		T4 (desufflation)	
**IL-6 Blood**	635 (226)		755 (254.8)		584 (176)		676.5 (207.5)	
*p*		***0.043 *** *(T1 vs. T2)*		*0.345 (T2 vs. T3)*		*1.00 (T3 vs. T4)*		
**IL-6 CSF**	611.5 (328.8)		917.5 (245.3)		822 (291)		825 (232)	
*p*		***0.028 *** *(T1 vs. T2)*		***0.043 *** *(T2 vs. T3)*		*0.715 (T3 vs. T4)*		
**TNFa Blood**	560 (798)		1070.5 (446.5)		710 (647)		854 (597)	
*p*		***0.012 *** *(T1 vs. T2)*		*0.499 (T2 vs. T3)*		*0.612 (T3 vs. T4)*		
**TNFa CSF**	118.5 (160.3)		245 (331)		195 (396.5)		284.5 (497.5)	
*p*		*0.068 (T1 vs. T2)*		*0.463 (T2 vs. T3)*		*0.345 (T3 vs. T4)*		
**Lac Blood**	0.1 (1.85)		1.15 (1.3)		1.15 (1.375)		1.15 (1)	
*p*		*0.752 (T1 vs. T2)*		*0.496 (T2 vs. T3)*		*0.6 (T3 vs. T4)*		
**Lac CSF**	1.4 (1.7)		2.3 (1.3)		1.6 (2.8)		1.9 (1.6)	
*p*		***0.017 *** *(T1 vs. T2)*		*0.237 (T2 vs. T3)*		*0.109 (T3 vs. T4)*		

CSF: cerebrospinal fluid; IL-6: interleukin 6, Lac: lactate, TNFa: tumor necrosis factor alpha. Data are displayed as median and interquartile range in parentheses. IL-6 and TNFa are expressed as *pg/ml *and lactate as *mmol/L*. Statistical significances (*p < 0.05*) are marked in bold. IAP, intra-abdominal pressure.

#### Interleukin 6

Abdominal insufflation to IAP 20 mmHg (T2) resulted in a statistically significant increase of IL-6 in blood (*p = 0.043*) and CSF (*p = 0.028*). Spearman correlation coefficient analysis demonstrated a statistically significant co-variance of changes of intraspinal pressure (ΔISP) and CSF IL-6 (ΔIL-6), *p = 0.042*, during this phase. Further increase of IAP to 45 mmHg was followed by a statistically significant decrease in IL-6 in CSF (*p = 0.043*), and, finally, abdominal desufflation was followed by an increase of IL-6.

#### Tumor necrosis factor alpha

TNFa was increased in both blood and CSF. However, a statistically significant (*p = 0.012*) increase of TNFa was demonstrated only in blood during an increase of IAP to 20 mmHg (T2). Further insufflation to 45 mmHg was followed by a slight decrease of TNFa concentrations in blood and CSF; finally, an increase of TNFa was observed after abdominal desufflation.

### Lactate (Table 1)

Lactate showed an increase in both blood and CSF after increase of IAP to 20 mmHg, with a statistically significant change (*p = 0.017*) demonstrated in the CSF. However, a further increase of IAP to 45 mmHg (T3) resulted in a decrease of CSF lactate, which was slightly increased after abdominal desufflation.

### Central nervous system pressures (Table 2)

**Table 2:** Changes of cerebral and spinal perfusion pressures during the four experimental phases (T1 to T4).

	T1 (baseline)		T2 (IAP = 20 mmHg)		T3 (IAP = 45 mmHg)		T4 (desufflation)	
**MAP**	85.8 ± 9.62		79.8 ± 11.7		86.8 ± 15.73		84.6 ± 7.37	
*p*		*0.186 (T1 vs. T2)*		*0.297(T2 vs. T3)*		*0.625 (T3 vs. T4)*		
**ICP**	18.7 ± 7.57		25.4 ± 7.79		26.8 ± 9.17		15.3 ± 3.65	
*p*		***<0.001*** *(T1 vs. T2)*		*0.485 (T2 vs. T3)*		***<0.001 *** *(T3 vs. T4)*		
**CPP**	67.1 ± 13.81		54.4 ± 9.77		60 ± 16.54		69.3 ± 8.38	
*p*		***0.013 *** *(T1 vs. T2)*		*0.392 (T2 vs. T3)*		*0.057 (T3 vs. T4)*		
**ISP**	13.2 ± 3.26		25.4 ± 8.36		22.3 ± 8		12.3 ± 3.86	
*p*		***0.001*** *(T1 vs. T2)*		*0.375 (T2 vs. T3)*		***0.005 *** *(T3 vs. T4)*		
**SPP**	72.6 ± 10.95		54.4 ± 10.6		64.5 ± 21.57		72.3 ± 7.42	
*p*		***0.002 *** *(T1 vs. T2)*		*0.198 (T2 vs. T3)*		*0.235 (T3 vs. T4)*		

CPP is calculated as mean arterial pressure (MAP) minus intracranial pressure (ICP), while SPP as MAP minus intraspinal pressure (ISP). All pressures are displayed as mean ± SD and expressed as mmHg. Statistical significances (*p < 0.05*) are marked with bold. IAP: intra-abdominal pressure.

Unexpectedly, baseline ICP and ISP were above normal levels (mean 18.7 and 13.2 mmHg, respectively). However, increase of IAP to 20 mmHg resulted in statistically significant (*p *< 0.001) further increases of ICP and ISP, whereas CPP (*p *= 0.013) and SPP (*p *= 0.002) decreased significantly under the threshold for ischemia of 60 mmHg [24]. Paradoxically, further increase of IAP to 45 mmHg was followed by a minor increase of ICP and a decrease of ISP, with a concomitant improvement of perfusion pressures above 60 mmHg. After abdominal desufflation all measurements returned to baseline levels.

#### Abdominal - CNS transmission

The transmission of the changes of IAP to another compartment as the cranial and spinal is assessed by the index of transmission [8], which is expressed as percentage and is calculated using the equation:

In order to compare the IAP in every phase we used the measurements of IVCP, as follows:

T1 = 9.9 mmHg, T2 = 20.4 mmHg, T3 = 44.1 mmHg and

T4 = 11.4 mmHg.

Thus, the index of transmission of IAP to CNS is the following:

a. IAP (T1 → T2): 63.8% (ICP), 116% (ISP),

b. IAP (T1 → T3): 23.6% (ICP), 26.6% (ISP).

### Cardiovascular system (Figures 1, 2, 3, 4 and 5)

**Figure 1:**
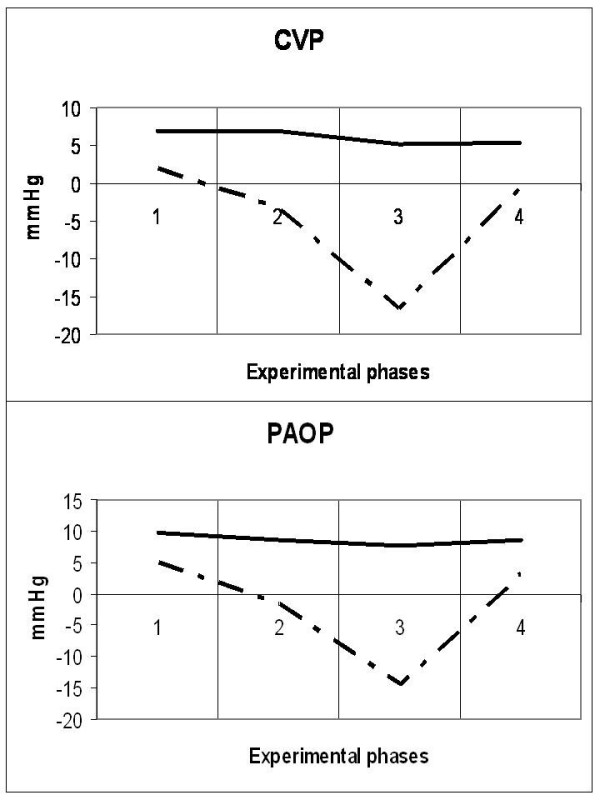
**Diagrammatic presentation of alterations of central venous pressure (CVP) and pulmonary artery occlusion pressure (PAOP)**. These changes occurred during the four experimental phases (1: baseline, 2: IAP 20 mmHg, 3: IAP 45 mmHg and 4: abdominal desufflation). Traditional measurements are depicted by the solid line and transmural measurements by the intermitted line.

**Figure 2:**
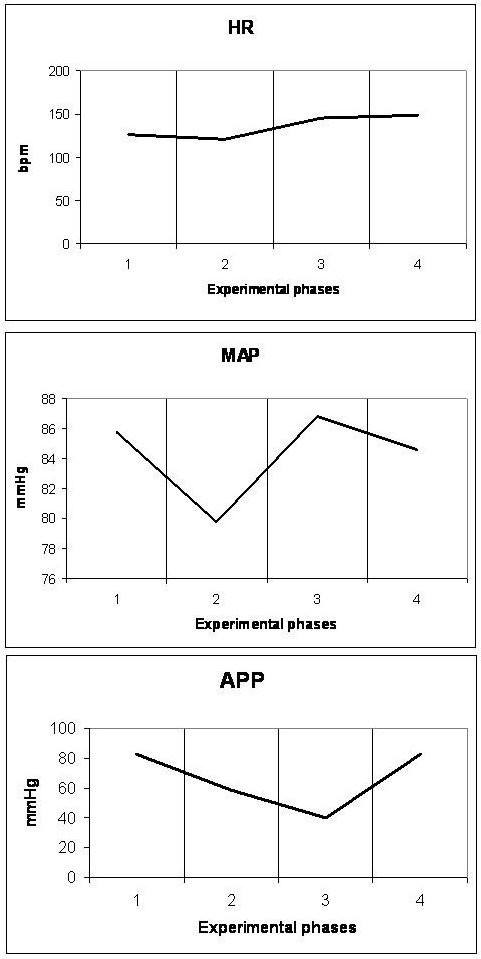
**Diagrammatic presentation of alterations of heart rate (HR), mean arterial pressure (MAP) and abdominal perfusion pressure (APP)**. These changes occurred during the four experimental phases (1: baseline, 2: IAP 20 mmHg, 3: IAP 45 mmHg and 4: abdominal desufflation).

**Figure 3:**
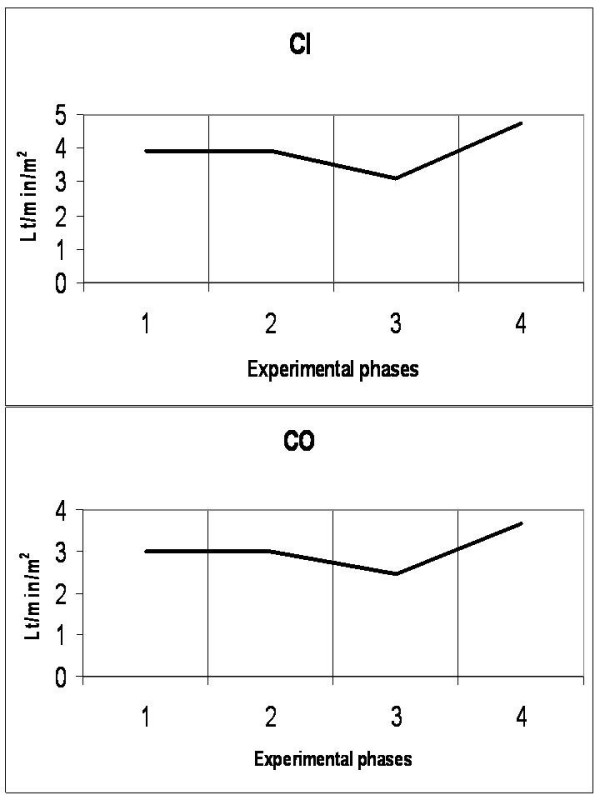
**Diagrammatic presentation of alterations of cardiac index (CI) and cardiac output (CO)**. These changes occurred during the four experimental phases (1: baseline, 2: IAP 20 mmHg, 3: IAP 45 mmHg and 4: abdominal desufflation).

**Figure 4:**
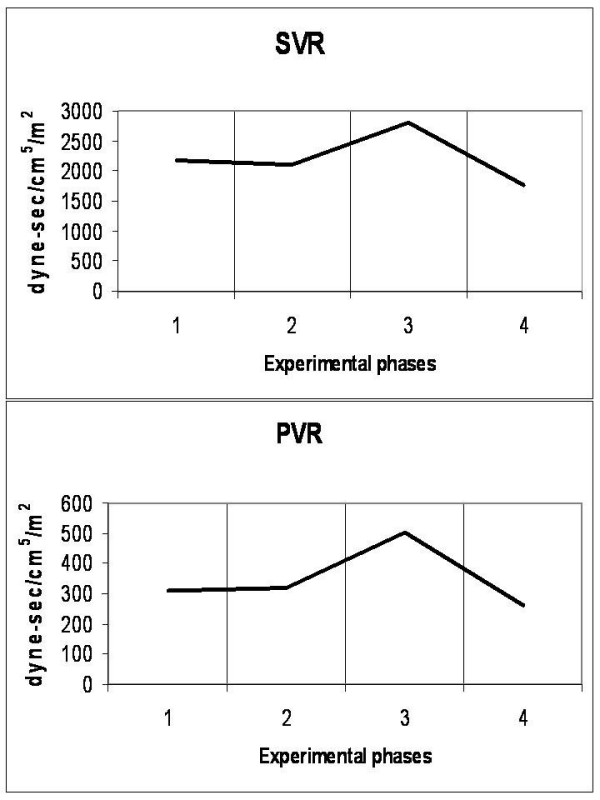
**Diagrammatic presentation of alterations of systemic (SVR) and pulmonary (PVR) vascular resistances**. These changes occurred during the four experimental phases (1: baseline, 2: IAP 20 mmHg, 3: IAP 45 mmHg and 4: abdominal desufflation).

**Figure 5:**
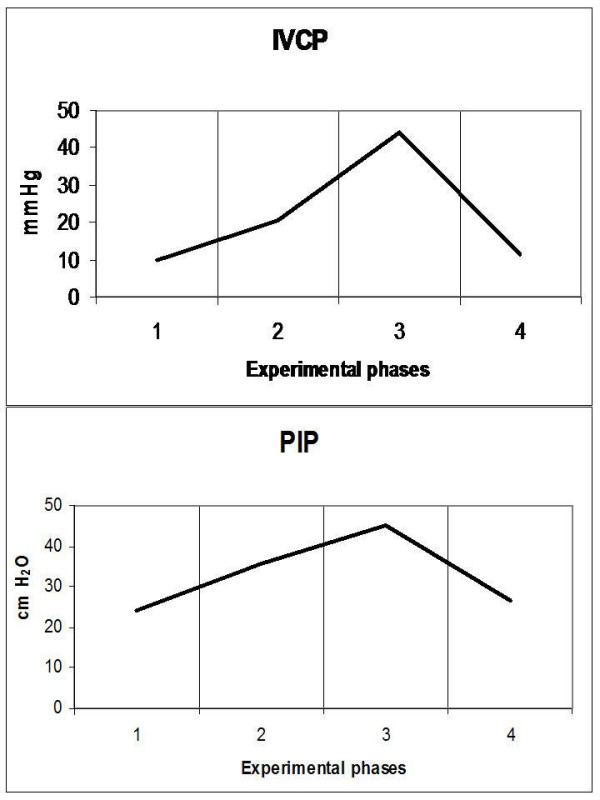
**Diagrammatic presentation of alterations of inferior vena cava pressure (IVCP) and peak inspiratory pressure (PIP)**. These changes occurred during the four experimental phases (1: baseline, 2: IAP 20 mmHg, 3: IAP 45 mmHg and 4: abdominal desufflation).

Animals were considered normovolemic due to estimation of the preload status with traditional CVP measurement which was misleading. Calculation of the transmural intracardiac filling pressures revealed that animals were hypovolemic with an associated tachycardia (Figure 1). Heart rate initially decreased in phase T2, but was increased in the two subsequent phases, with a parallel increase in MAP and a decrease of APP (Figure 2). Cardiac output and cardiac index were decreased in phase T3 and restored to baseline levels after abdominal desufflation (Figure 3). Systemic and pulmonary vascular resistances increased significantly with IAH and decreased after abdominal desufflation (Figure 4). IVCP reflected accurately the changes in IAP (Figure 5).

### Respiratory function and acid-base homeostasis (Figures 5, 6, 7)

**Figure 6:**
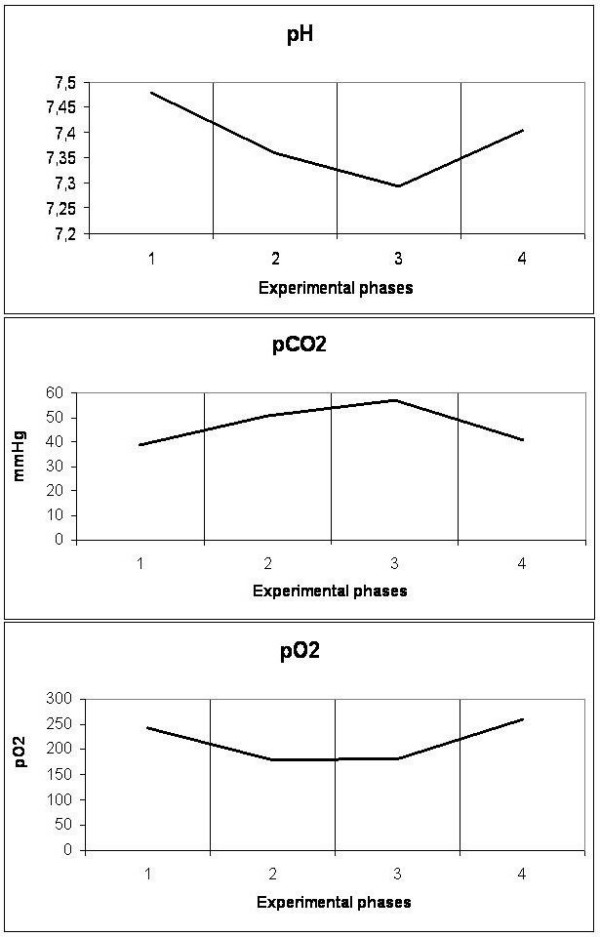
**Diagrammatic presentation of alterations of pH, pCO_2 _and pO_2_**. These changes occurred during the four experimental phases (1: baseline, 2: IAP 20 mmHg, 3: IAP 45 mmHg and 4: abdominal desufflation).

**Figure 7:**
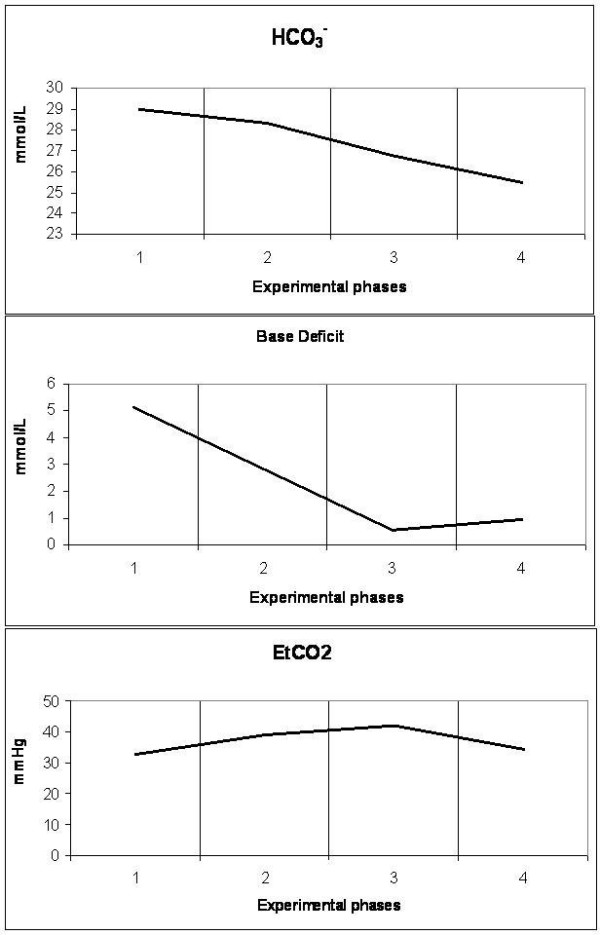
**Diagrammatic presentation of alterations of bicarbonate (HCO_3_^-^), base deficit and end-tidal carbon dioxide (EtCO_2_)**. These changes occurred during the four experimental phases (1: baseline, 2: IAP 20 mmHg, 3: IAP 45 mmHg and 4: abdominal desufflation).

IAH increased peak inspiratory pressure (PIP) in both phases T2 and T3, declining after abdominal desufflation (Figure 5). In contrary, pH was decreased during T2 and T3 and increased to baseline levels after removal of the pneumoperitoneum (Figure 6). This change was associated with an increase in pCO_2 _in the same phases, which returned to baseline levels after desufflation. A concomitant decrease in bicarbonate and base deficit were observed, without, in fact, compensating the acute respiratory acidosis (Figure 7). Finally, end-tidal carbon dioxide (EtCO_2_) varied between pre-established limits (35 to 45 mmHg) (Figure 7).

## Discussion

In the present study, we analyzed the changes in CNS perfusion pressures in relation to changes in CSF pro-inflammatory cytokines (IL-6 and TNFa) and a metabolite (lactate), during a controlled increase in IAP, in order to determine whether CNS ischemia ensued. The main findings of this experimental study are as follows: first, all ischemic mediators (IL-6, TNFa and lactate) were significantly increased when both perfusion pressures (CPP and SPP) decreased less than 60 mmHg; second, IL-6 was considered the most sensitive marker of ISP rise; third, all ischemic mediators decreased when perfusion pressures increased more than 60 mmHg, irrespective of the level of IAH and, finally, IAH had a negative impact on cardio-respiratory function and acid-base homeostasis.

According to the guidelines for the management of severe traumatic brain injury of the Brain Trauma Foundation [38], hypotension (systolic blood pressure <90 mmHg), hypoxia (PaO_2 _<60 mmHg or O_2 _saturation <90%), ICP >20 mmHg and CPP < 60 mmHg should be avoided, in order to prevent cerebral ischemia. In our experimental study hypotension and hypoxia were not observed. However, CNS perfusion pressures (CPP and SPP) were both decreased lower than the ischemia threshold of 60 mmHg when the IAP was increased to 20 mmHg (phase T2). This decrease was concomitantly associated with a significant increase of ischemic mediators. Both changes indicate that CNS ischemia ensued. The significant statistical correlation of the changes of ISP and IL-6 measured in the CSF (ΔISP vs. ΔIL-6_csf_) concludes that IL-6 is a more sensitive marker of ISP changes than the other two mediators.

A limitation of this study is that modern technology was not used for practical reasons. Modern technology uses multiparametric neuromonitoring to support brain trauma victims in current clinical practice by inserting intracranially probes that can measure directly intracranial pressure, brain tissue oxygenation, vascular flow and cerebral metabolism [39-43]. Despite the absence of more direct methods of CNS ischemia demonstration, the suggestion that ischemia ensued in phase T2 (IAP 20 mmHg) was reinforced by the observation in phase T3 (IAP 45 mmHg): CNS perfusion pressures increased more than the ischemia threshold of 60 mmHg, which was followed by a decrease in all ischemia indicators, irrespective of the presence of even higher IAP. Improvement of CNS perfusion was a result of an increase of MAP, which in turn resulted from an augmented compensatory tachycardia, due to further decrease in preload parameters. An alternative explanation for increased MAP is provided by Citerio G et al [20] who state that increased intrathoracic pressure facilitates systolic ejection. Another possible explanation of this phenomenon could be that the first period of elevated CNS pressure and ischemia (phase T2) acted as a preconditioning period alleviating further ischemic changes of the brain during phase T3 [44-48].

Another interesting point is that a further increase of IAP to 45 mmHg was not followed by a similar dramatic increase of ICP and ISP. On the contrary, ISP decreased and ICP was only slightly increased. This phenomenon is explained by the reduction of the CSF volume by sampling: approximately 0.5 to 1 ml of CSF aspirated in every experimental phase, multiplied by three (T1 to T3) is 1.5 to 4.5 ml of CSF withdrawn. A clinical paradigm of this effect is the prevention of paraplegia with CSF drainage during surgical repair of extended thoracoabdominal aneurysms [49,50], in order to alleviate intraspinal hypertension.

Abdominal desufflation (phase T4) was followed by restoration of CNS pressures to baseline levels and a further increase of all indicators (IL-6, TNFa and lactate), the latter resulting probably due to systematic and CNS reperfusion.

The negative impact of IAH on the cardiovascular system (decreased preload, decreased contractility, increased afterload), airway pressure (increased PIP) and acid-base homeostasis (acute respiratory acidosis) is in accordance with the currently described and accepted pathophysiological implications of the syndrome [3-5,7,37]. Abdominal desufflation was followed by normalization of all these parameters.

However, this study had many inherent limitations. The small sample size, the absence of controls and any intervention, and the high IAP used in phase T3 (45 mmHg, not extrapolated in the clinical setting) are inherent parameters confined *a priori *by the experimental protocol. Other important limitations which interfered with data measurements and interpretation are the following:

### Hypovolemia

During this experimental study traditional measurements of CVP and PAOP were used for the assessment of the preload status. According to them, all animals were normovolemic. However, after collecting data and calculating the transmural pressures, we realized that all animals were actually hypovolemic.

a) Transmural CVP (mmHg): 1.9 (T1)/-3.3 (T2)/-16.6 (T3)/-0.5 (T4)

b) Transmural PAOP (mmHg): 4.85 (T1)/-1.6 (T2)/-14.4 (T3)/3 (T4).

This observation (hypovolemia) explains the unexpected baseline tachycardia (which was attributed initially to not adequate depth of anaesthesia or administration of atropine, and so on). Moreover, this is a condition that augments the impact of IAH on the cardiovascular system.

### High baseline IAP

Changes in IVCP are known to reflect accurately changes in IAP [3]. This was actually confirmed in our study. However, we observed that baseline IAP was increased at the beginning (9.9 mmHg) and at the end of the experiment (11.4 mmHg). An explanation of this is provided by the mechanism of action of fentanyl, administered for maintenance of anaesthesia: fentanyl induces muscle contraction and rigidity of the chest and abdominal wall, as well as the extremities above a critical concentration [51-55].

### High baseline ICP

Moderately increased baseline IAP was responsible for a similar moderate increase of ICP, according to the mechanisms that have been proposed by Bloomfield and Halverson [13-15].

The two last limitations (high IAP and ICP) resemble the clinical scenario of development of IAH in patients with the presence of already increased ICP (due to trauma, vascular accidents or metabolic causes).

## Conclusions

IAH significantly reduces cerebral and spinal perfusion pressures, concomitantly increasing IL-6, lactate and TNFa in CSF, suggesting the development of CNS ischemia. However, this effect was transient and reversible when perfusion pressures were restored to a level above 60 mmHg, irrespective of the level of IAH.

## Key messages

• Intra-abdominal hypertension led to increases of ICP and ISP.

• Increased ICP and ISP resulted in decreases of CPP and SPP, respectively.

• When CPP and SPP decreased below 60 mmHg an increase in IL-6, TNFa and lactate in the CSF suggested the development of CNS ischemia

## Abbreviations

ACS: abdominal compartment syndrome; CNS: central nervous system; CVP: central venous pressure; CPP: cerebral perfusion pressure; CSF: cerebrospinal fluid; EtCO_2_: end-tidal carbon dioxide; GEDV: global end-diastolic volume; IVCP: inferior vena cava pressure; IL-6: interleukin 6; IAH: intra-abdominal hypertension; IAP: intra-abdominal pressure; ICP: intracranial pressure; ISP: intraspinal pressure; IQR: interquartile range; ITP: intrathoracic pressure; MAP: mean arterial pressure; PAOP: pulmonary artery occlusion pressure; PIP: peak inspiratory pressure; RVEDV: right ventricular end-diastolic volume; SPP: spinal perfusion pressure; SVV: stroke volume variation; TNFa: tumor necrosis factor.

## Competing interests

The authors declare that they have no competing interests.

## Authors' contributions

AM, EA and DV designed the study; AM, EA, PV, GP and EB conducted the experiments. PB and KT statistically analyzed the results. AM and EA drafted the manuscript; AM, EA, EB and DV critically revised the manuscript.
